# Artificial intelligence-based detection of paediatric appendicular skeletal fractures: performance and limitations for common fracture types and locations

**DOI:** 10.1007/s00247-023-05822-3

**Published:** 2023-12-15

**Authors:** Irmhild Altmann-Schneider, Christian J. Kellenberger, Sarah-Maria Pistorius, Camilla Saladin, Debora Schäfer, Nidanur Arslan, Hanna L. Fischer, Michelle Seiler

**Affiliations:** 1 Department of Diagnostic Imaging, University Children’s Hospital Zurich, Steinwiesstrasse 75, 8032 Zurich, Switzerland; 2 Paediatric Emergency Department, University Children’s Hospital Zurich, Steinwiesstrasse 75, 8032 Zurich, Switzerland; 3 Children’s Research Centre, University Children’s Hospital Zurich, Steinwiesstrasse 75, 8032 Zurich, Switzerland

**Keywords:** Artificial intelligence, Appendicular skeleton, Fracture, Paediatric, Radiograph

## Abstract

**Background:**

Research into artificial intelligence (AI)-based fracture detection in children is scarce and has disregarded the detection of indirect fracture signs and dislocations.

**Objective:**

To assess the diagnostic accuracy of an existing AI-tool for the detection of fractures, indirect fracture signs, and dislocations.

**Materials and methods:**

An AI software, BoneView (Gleamer, Paris, France), was assessed for diagnostic accuracy of fracture detection using paediatric radiology consensus diagnoses as reference. Radiographs from a single emergency department were enrolled retrospectively going back from December 2021, limited to 1,000 radiographs per body part. Enrolment criteria were as follows: suspected fractures of the forearm, lower leg, or elbow; age 0–18 years; and radiographs in at least two projections.

**Results:**

Lower leg radiographs showed 607 fractures. Sensitivity, specificity, positive predictive value (PPV), and negative predictive value (NPV) were high (87.5%, 87.5%, 98.3%, 98.3%, respectively). Detection rate was low for toddler’s fractures, trampoline fractures, and proximal tibial Salter-Harris-II fractures. Forearm radiographs showed 1,137 fractures. Sensitivity, specificity, PPV, and NPV were high (92.9%, 98.1%, 98.4%, 91.7%, respectively). Radial and ulnar bowing fractures were not reliably detected (one out of 11 radial bowing fractures and zero out of seven ulnar bowing fractures were correctly detected). Detection rate was low for styloid process avulsions, proximal radial buckle, and complete olecranon fractures. Elbow radiographs showed 517 fractures. Sensitivity and NPV were moderate (80.5%, 84.7%, respectively). Specificity and PPV were high (94.9%, 93.3%, respectively). For joint effusion, sensitivity, specificity, PPV, and NPV were moderate (85.1%, 85.7%, 89.5%, 80%, respectively). For elbow dislocations, sensitivity and PPV were low (65.8%, 50%, respectively). Specificity and NPV were high (97.7%, 98.8%, respectively).

**Conclusions:**

The diagnostic performance of BoneView is promising for forearm and lower leg fractures. However, improvement is mandatory before clinicians can rely solely on AI-based paediatric fracture detection using this software.

**Graphical Abstract:**

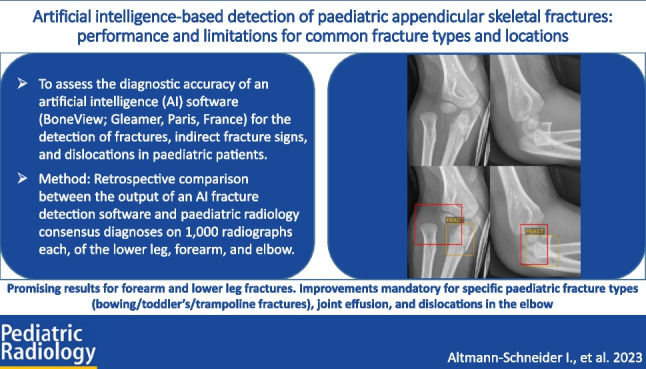

## Introduction

In recent years, artificial intelligence (AI)-based software applications have rapidly advanced in radiology across various subspecialties and are a subject of extensive research. Automated fracture detection on conventional radiographs is one of the applications for which several AI-based software packages have been developed, mainly for the adult patient population [1]. For the paediatric population, progress in this regard lags behind. The morphological changes of the developing skeleton, normal variants during growth, distinct paediatric fracture types and the changing fracture patterns pose significant challenges for AI-based software development. Additionally, smaller patient populations hamper the creation of large databases, which are necessary to train the software sufficiently. Despite growing interest in AI-based software applications for automated paediatric fracture detection, research in this area remains scarce [2]. Most studies have focused on one specific body part and have described the development and evaluation of the software in a single-centre setting [3–5]. A few studies have performed external assessment of AI aids for fracture detection, however mostly with relatively small numbers of radiographs per anatomic region. Existing research into AI-based fracture detection in the paediatric patient population has so far disregarded the detection of indirect fracture signs and dislocations. Detection of paediatric fractures is often challenging, partly because they may be occult. This emphasizes the critical importance of detecting indirect fracture signs, especially in the elbow joint, where an effusion might be the only indicator of a fracture.

We aimed to assess the diagnostic accuracy of an existing AI tool for the detection of relevant paediatric posttraumatic effects, including fractures, indirect fracture signs, and dislocations, using a large dataset comprising the three most common paediatric fracture locations, the lower leg, forearm, and elbow.

## Materials and methods

### Ethics approval

This research has been carried out in accordance with the Code of Ethics of the World Medical Association (Declaration of Helsinki) and the study has been approved by the cantonal research ethics committee (2022–00816).

### Data acquisition and subjects

In this study, consecutive radiographs of patients, ages 0–18 years, who attended the emergency department of the University Children’s Hospital, Zurich due to an acute trauma and suspected fracture, were enrolled retrospectively going back from December 2021. Inclusion criteria were radiographs of either the forearm, lower leg, or elbow in at least two projections (frontal and lateral), performed on the day of attendance to the emergency department. Radiographs of patients with fractures with a high specificity for child abuse (e.g., metaphyseal corner fractures) were excluded as the AI software is not trained to detect them.

A total of 1,000 patients were enrolled for each body part (inclusion ended in December 2017 for lower leg radiographs, in May 2020 for forearm radiographs, and in August 2019 for elbow radiographs). Paired bones in lower leg and forearm images were analyzed separately counting as separate cases. In the case of more than one posttraumatic change per bone, then each finding was counted as a separate case. Each elbow joint was counted as one case. In the case of more than one posttraumatic change in the elbow, then each finding was counted as a separate case.

Radiographs were obtained on two different radiographic machines (Philips, Best, The Netherlands, and Siemens Healthcare, Erlangen, Germany).

### Reference standard and readers

All radiographs contained an original report from the attending paediatric radiology staff member with varying degrees of experience. Each radiograph received a second reading performed by a certified paediatric radiologist with three (I.A-S. and S.P.) and seven (C.S.) years of experience, who had access to the clinical information. Disagreements between the original report and the second reading were resolved by a third consensus reading (I.A-S., S.P., and C.S.). This paediatric radiology consensus diagnosis was used as reference standard for assessing the diagnostic accuracy of the AI software. All fractures were classified according to the paediatric AO (Arbeitsgemeinschaft für Osteosynthesefragen) classification [6] and were further analyzed when ten or more cases of the same type were detected. For image interpretation, Sectra workstations (IDS7) (Sectra AB, Linköping, Sweden) were used.

### Artificial intelligence software

BoneView™ (Gleamer, Paris, France) is a commercially available AI-based computer-aided diagnosis tool. It is a CE-marked medical device designed for the detection of acute and subacute fractures, effusions, dislocations, and focal bone lesions on full resolution Digital Imaging and Communications in Medicine images, with additional US Food and Drug Administration clearance for paediatric fracture detection. The algorithm uses a deep convolutional neural network based on the object detection framework (“Detectron 2”) that works as a two-stage object detector. The algorithm works with two operating points: doubt when the confidence score lies between 50% and 90% and positive when the confidence score exceeds 90%. A confidence score between 0% and 50% will be annotated as negative. These operating points were chosen based on the free response operating characteristic curve of the development test set to achieve a high sensitivity and a high specificity operating point. The algorithm was developed using a dataset of more than 300,000 radiographs from more than 60 radiology departments collected from January 2011 to May 2023. The dataset for development was randomly split into 70% for training, 10% for validation, and 20% for internal test sets without any overlap of patients or images between the subsets.

### Statistical analyses

Statistical Package for Social Sciences software for Windows (IBM, version 29.0, Armonk, NY) was used for statistical analyses. Sensitivity, specificity, positive predictive value (PPV), and negative predictive value (NPV) were calculated for fracture detection in each of the three anatomical regions to assess the diagnostic performance of the AI software. These values were also calculated for joint effusion and dislocations on elbow radiographs. The statistical analyses were conducted twice, once considering cases annotated with doubt (confidence score on both projections between 50% and 90%) as true fractures and once categorising them as no fractures. In the case of a classification disagreement between projections, the case was classified by the finding of highest confidence.

## Results

A total of 1,000 patients were included in the study for each anatomical location. Mean age was 7.8 ± 3.9 years for forearm radiographs (60% male) and 7.7 ± 3.7 years for elbow radiographs (55% male). For lower leg radiographs, the mean age was slightly lower, 4.9 ± 4 years (58% male). Detailed characteristics of the study population are shown in Table 1.

**Table 1:** Patient characteristics

Patient characteristic	Lower leg	Forearm	Elbow
Mean age ± SD	4.9 ± 4	7.8 ± 3.9	7.5 ± 3.7
Sex			
Females, *n*	422	400	453
Males, *n*	578	600	547

*SD* standard deviation

### Lower leg radiographs

**Fig. 1:**
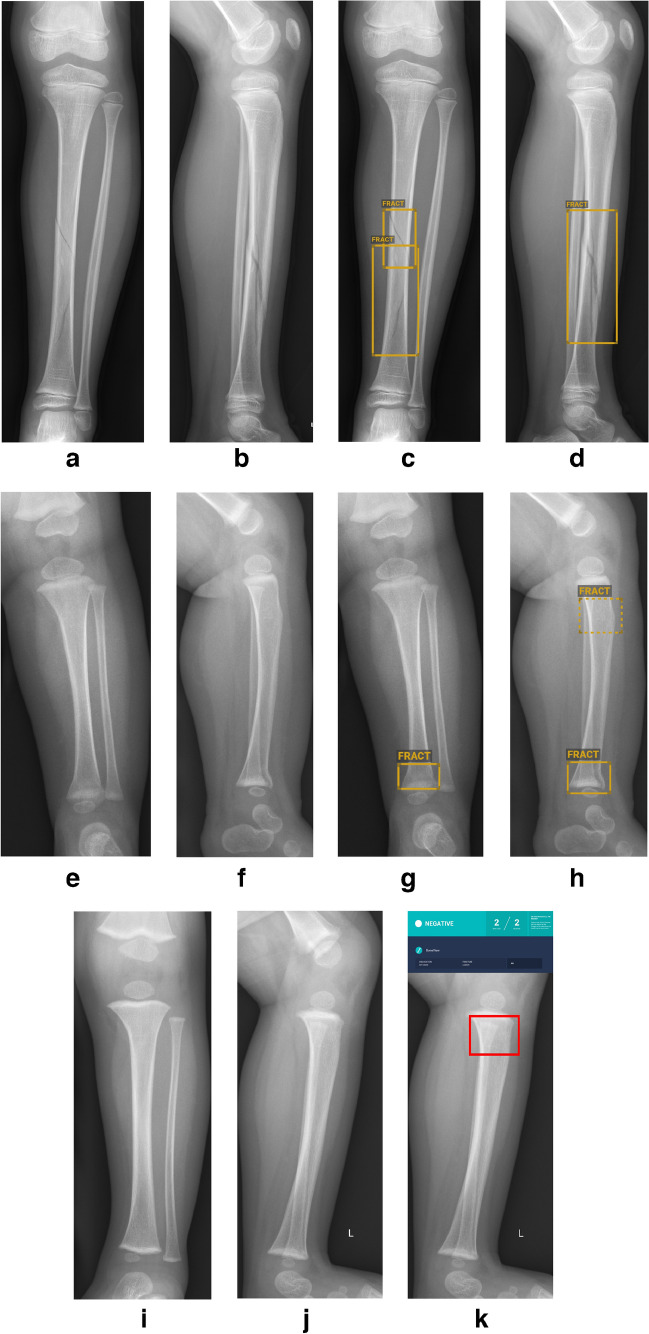
Examples of lower leg radiographs showing true positive (**a–d**), false positive (**e–h**) and false negative (**i–k**) diagnoses made by BoneView (Gleamer, Paris, France). **a–d** Anteroposterior (AP) and lateral radiographs of the left lower leg in a 5-year-old girl before (**a,b**) and after (**c,d**) annotation by BoneView. There is a tibial spiral fracture. The fracture was detected with a confidence score above 90% (*rectangles/“FRACT”*). **e–h** AP and lateral radiographs of the left lower leg in an 11-month-old girl before (**e,f**) and after (**g,h**) annotation by BoneView. There is a distal tibial Salter-Harris-II fracture. The true positive finding of a fracture was detected with a confidence score above 90% (*rectangles/“FRACT*”). Additionally, BoneView detected a false positive proximal fracture on the lateral radiograph (**h**) with a confidence score between 50% and 90% (*broken rectangle/“FRACT”*). **i–k** AP and lateral radiographs of the left lower leg in a 1-year-old boy before (**i,j**) and after (**k**) annotation by BoneView. There is a proximal metaphyseal tibial buckle fracture (trampoline fracture), which is only visible on the lateral radiograph (*rectangle*). BoneView did not detect the fracture

A total of 2,100 cases were included, with 607 confirmed fractures, comprising 142 (23%) fibular and 465 (77%) tibial fractures. Details are shown in Table 2. Classifying doubt cases as fractures, sensitivity, specificity, PPV, and NPV all exceeded 90%. Classifying doubt cases as no fractures then only a slight drop in sensitivity below 90% was observed (Table 3). Detection rates for specific fracture types are shown in Table 4. Detection rates were below 80% for nondisplaced spiral fractures of the tibial diaphysis (toddler´s fractures), proximal tibial impaction fractures (trampoline fractures), and Salter-Harris-II fractures of the proximal tibia. Figure 1 illustrates examples of true positive, false positive, and false negative cases.

**Table 2:** Radiograph characteristics per anatomical region

	Lower leg	Forearm	Elbow
Radiographs, *n*	1,000	1,000	1,000
Cases, *n*	2,100	2,051	1,104
Radius/ulna, *n*		1,019/1,032	
Fibula/tibia, *n*	1,049/1,051		
Fractures, *n*	607	1137	517
Humerus, *n*			397
Radius/ulna, *n*		673/464	69/51
Fibula/tibia, *n*	142/465

**Table 3:** Lower leg radiographs

Statistic	Doubt cases=fracture	Doubt cases=no fracture
Sensitivity (95% CI)	90.6 (88.0–92.8)	87.4 (84.6–90.0)
Specificity (95% CI)	97.1 (96.1–97.9)	99.4 (98.9–99.7)
Positive predictive value	92.6	98.3
Negative predictive value	96.2	95.1

*CI* confidence interval

**Table 4:** Lower leg fracture types with frequency and detection rate

Fracture (#) type	Number of fractures/frequency (%)	Doubt/false negatives	Detection rate (%)
Oblique diaphyseal # tibia	149/25	0/0	100
Toddler`s #	66/11	3/14	74
Buckle # proximal tibia (trampoline fracture)	50/8	6/11	66
Buckle # distal tibia	46/8	0/1	98
Salter-Harris-II # distal tibia	32/5	0/6	81
Buckle # distal fibula	31/5	1/5	81
Complete metaphyseal # distal tibia	30/5	0/1	97
Complete metaphyseal # proximal tibia	25/4	0/0	100
Complete diaphyseal # tibia	24/4	0/0	100
Complete diaphyseal # fibula	22/4	1/1	91
Complete metaphyseal # distal fibula	19/3	1/2	84
Oblique diaphyseal # fibula	17/3	0/0	100
Greenstick # fibula	13/2	1/1	85
Salter-Harris-II # proximal tibia	10/2	1/3	60

### Forearm radiographs

**Fig. 2:**
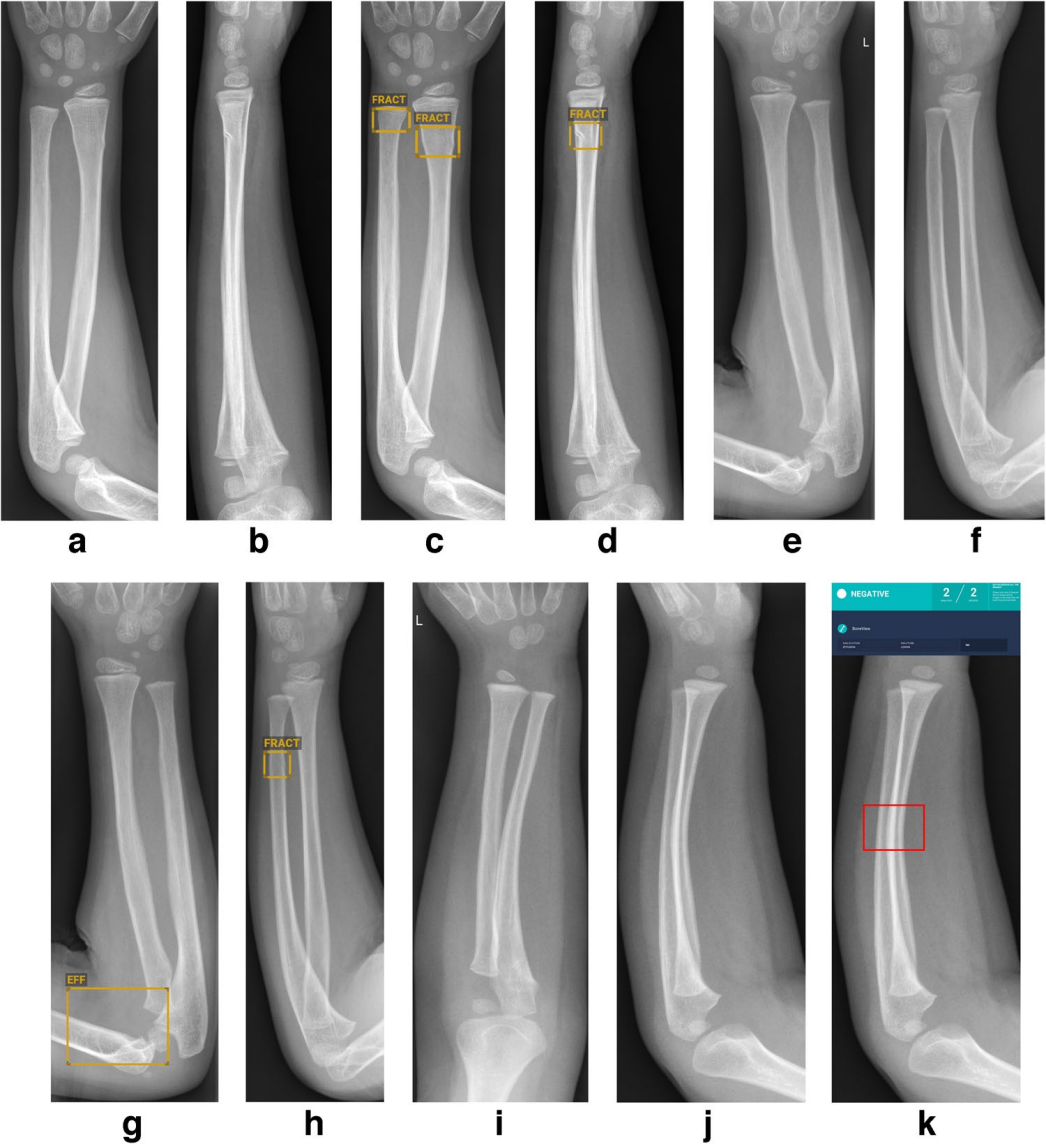
Examples of forearm radiographs showing true positive (**a–d**), false positive (**e–h**) and false negative (**i–k**) diagnoses made by BoneView (Gleamer, Paris, France). **a–d** Anteroposterior (AP) and lateral radiographs of the left forearm in a 5-year-old boy before (**a,b**) and after (**c,d**) annotation by BoneView. There are distal metaphyseal radial and ulnar buckle fractures. The fractures were detected with a confidence score above 90% (*rectangles/“FRACT”*). **e–h** AP and lateral radiographs of the left forearm in a 5-year-old boy before (**e,f**) and after (**g,h**) annotation by BoneView. The radiographs were normal. BoneView detected a false positive distal diaphyseal ulnar fracture on the lateral radiograph with a confidence score above 90% (*rectangle/“FRACT”*). Additionally, false positive effusion in the elbow joint was detected (*rectangle/“EFF”*). **i–k** AP and lateral radiographs of the left forearm in a 2-year-old boy before (**i,j**) and after (**k**) annotation by BoneView. There is a radial bowing fracture which is only visible on the lateral radiograph (*rectangle*). BoneView did not detect the fracture

A total of 2,051 cases were included, with 1,137 confirmed fractures, comprising 673 (59%) radial and 464 (41%) ulnar fractures. Details are shown in Table 2. Classifying doubt cases as either fractures or no fractures, sensitivity, specificity, PPV, and NPV all exceeded 90% (Table 5). Detection rates for specific fracture types are summarized in Table 6. Detection rates were poor for bowing fractures (one out of 11 radial bowing fractures and zero out of seven ulnar bowing fractures were correctly detected). Additionally, the detection rate was below 80% for styloid process avulsions, proximal radial buckle fractures, and complete proximal ulnar fractures. Figure 2 illustrates examples of true positive, false positive, and false negative cases.

**Table 5:** Forearm radiographs

Statistic	Doubt cases = fracture	Doubt cases = no fracture
Sensitivity (95% CI)	96.0 (94.7–97.1)	92.9 (91.2–94.3)
Specificity (95% CI)	92.9 (91.0–94.4)	98.1 (97.0–98.9)
Positive predictive value	94.4	98.4
Negative predictive value	94.9	91.7

*CI* confidence interval

**Table 6:** Forearm fracture types with frequency and detection rate

Fracture (#) type	Number of fractures/frequency (%)	Doubt/false negatives	Detection rate (%)
Buckle # distal radius	263/23	4/1	98
Buckle # distal ulna	165/15	14/9	86
Complete metaphyseal # distal radius	132/12	1/0	99
Complete diaphyseal # radius	94/8	0/0	100
Complete diaphyseal # ulna	72/6	0/0	100
Complete metaphyseal # distal ulna	70/6	0/1	99
Greenstick # radius	56/5	0/2	96
Salter-Harris-II # distal radius	46/4	0/2	96
Oblique diaphyseal # ulna	44/4	0/0	100
Greenstick # ulna	37/3	0/1	97
Avulsion # ulnar styloid process	30/3	6/6	60
Oblique diaphyseal # radius	28/2	0/0	100
Buckle # proximal radius	20/2	3/4	65
Complete metaphyseal # proximal ulna	12/1	2/1	75
Bowing # radius	11/1	1/9	18
Complete metaphyseal # radial neck	10/1	1/1	80
Bowing # ulna	7/1	1/6	0

### Elbow radiographs

A total of 1,104 cases were included with 517 confirmed fractures, comprising 397 (77%) humeral, 69 (13%) radial, and 51 (10%) ulnar fractures. Details are shown in Table 2. Classifying doubt cases as fractures, sensitivity and NPV were high (91.5%, 89.5%, respectively), but specificity and PPV were low (63.7%, 69.0%, respectively). Classifying doubt cases as no fractures, then a remarkable decrease in sensitivity (80.5%) and increase in specificity (94.9%) and PPV (93.3%) was observed. BoneView frequently identified the humerus as a potential fracture site (doubt) in cases with significant joint effusion (possibly indicating occult fractures). A summary of the results is provided in Table 7. In elbow cases, the fracture detection rate exceeded 80% only for complete supracondylar fractures and complete fractures of the radial neck (Table 8).

**Table 7:** Elbow radiographs

Statistic	Doubt cases = fracture	Doubt cases = no fracture
Sensitivity (95% CI)	91.5 (88.7–93.8)	80.5 (76.8–83.8)
Specificity (95% CI)	63.7 (59.7–67.6)	94.9 (92.8–96.5)
Positive predictive value	69.0	93.3
Negative predictive value	89.5	84.7

*CI* confidence interval

**Table 8:** Elbow fracture types with frequency and detection rates

Fracture (#) type	Number of fractures/frequency (%)	Doubt/false negatives	Detection rate (%)
Complete supracondylar humerus #	249/48	5/0	98
Buckle # supracondylar humerus	53/10	11/0	76
Complete # proximal ulna (olecranon)	41/8	3/10	68
Complete # radial condyle with metaphyseal wedge (Salter-Harris-IV #)	39/8	6/4	74
Complete # radial neck	24/5	2/0	92
Buckle # proximal radius	21/4	5/6	48
Avulsion # ulnar epicondyle	16/3	5/7	25
Complete # ulnar condyle with metaphyseal wedge (Salter-Harris-IV #)	14/3	4/1	64
Salter-Harris-II # proximal radius	14/3	3/0	79

**Fig. 3:**
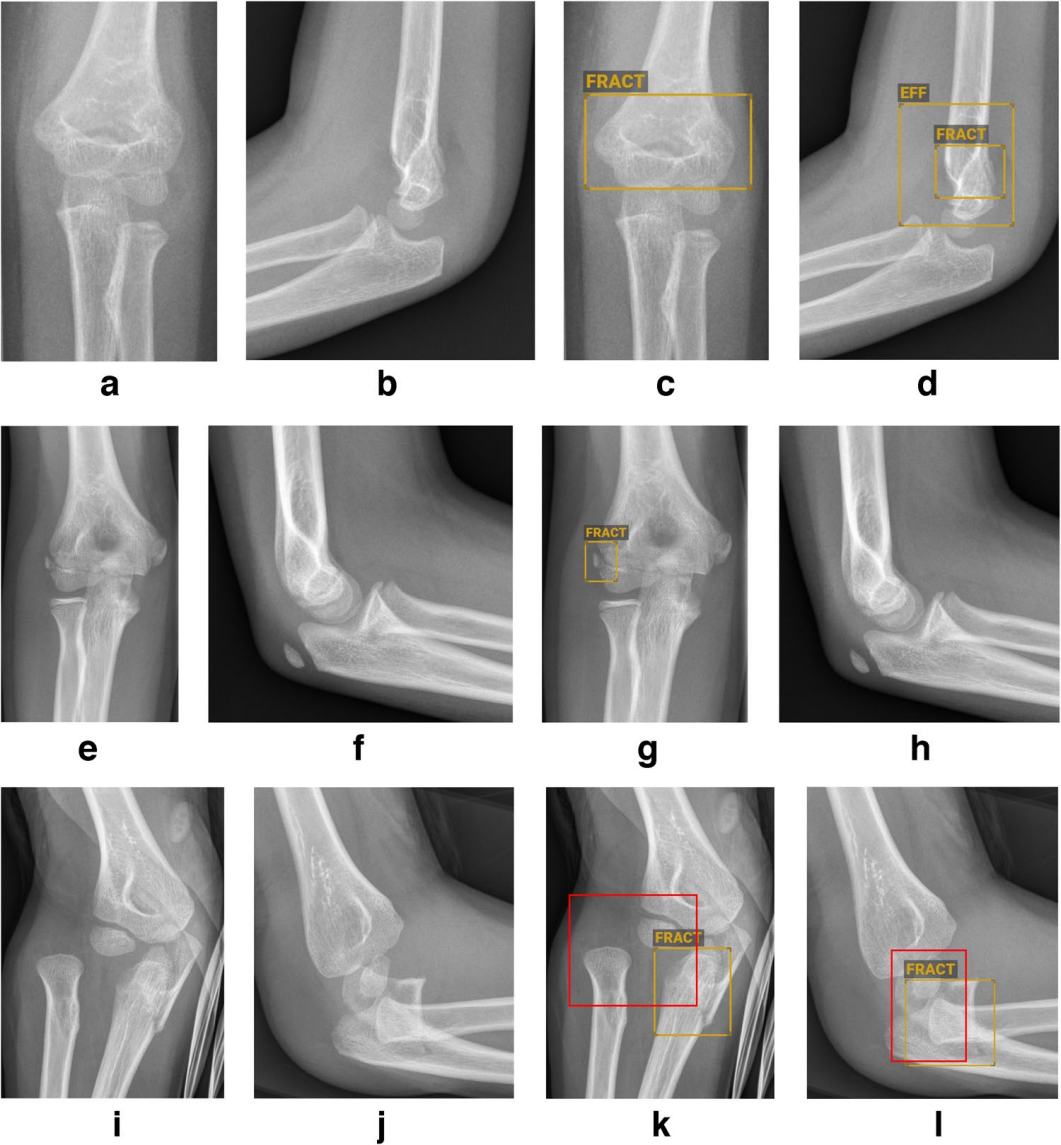
Examples of elbow radiographs showing true positive (**a–d**), false positive (**e–h**) and false negative (**i–k**) diagnoses made by BoneView (Gleamer, Paris, France). **a–d** Anteroposterior (AP) and lateral radiographs of the left elbow in a 4-year-old girl before (**a,b**) and after (**c,d**) annotation by BoneView. There is a complete supracondylar fracture with effusion (positive anterior and posterior fat pad sign). The fracture was detected with a confidence score above 90% (*rectangles/“FRACT”*). The effusion was depicted correctly by BoneView (*rectangle/“EFF”*). **e–h** AP and lateral radiographs of the right elbow in a 10-year-old girl before (**e,f**) and after (**g,h**) annotation by BoneView. The radiographs were normal. BoneView detected an avulsion of the radial epicondyle with a confidence score above 90% (*rectangle/“FRACT”*). **i–l** AP and lateral radiographs of the right elbow in a 6-year-old boy before (**i,j**) and after (**k**) annotation by BoneView. There is a Monteggia fracture-dislocation. BoneView detected the true positive complete fracture of the olecranon with a confidence score above 90% (*orange rectangle/“FRACT”*). The associated proximal radial dislocation was disregarded (*red rectangle*)

Overall, detection of joint effusion sensitivity was 85.1% with a specificity of 85.7%, a PPV of 89.5%, and NPV of 80%. In a subset of elbow cases without an obvious fracture, sensitivity and specificity slightly increased to 91.2% and 87.1%, respectively, while the PPV decreased to 78.8% and NPV increased to 95.0%. For the detection of elbow dislocations, sensitivity was 65.8% with a specificity of 97.7%, a PPV of 50%, and a NPV of 98.8%. Figure 3 illustrates examples of true positive, false positive, and false negative cases.

## Discussion

We evaluated the diagnostic accuracy of an AI-based software for automated fracture detection in a large paediatric patient cohort focussing on the three most common paediatric fracture locations.

Our evaluation of forearm fractures yielded promising results, in line with previously published data using the same AI software, demonstrating overall a high sensitivity, specificity, PPV, and NPV for fracture detection by the AI software as a stand-alone [7, 8]. In our study, all ulnar bowing fractures (*n*=7) and ten out of 11 radial bowing fractures were not detected. Bowing fractures are among the most difficult paediatric fractures to detect and studies show higher interrater variability even among paediatric radiologists as some bowing of the radius and ulna can still be physiological [9]. Moreover, detection rates for avulsions of the ulnar styloid process, buckle fractures of the proximal radius, and complete fractures of the proximal ulna (olecranon) were below 80%. Prior research has not explored different fracture types and sites per bone in such detail. The lower performance for these specific fracture types may be partly attributed to the low incidence, limiting the effectiveness of the training algorithms.

The evaluation of lower leg fractures showed overall promising results, but indicated areas for improvement. We noted relatively low detection rates for toddler’s fractures (74%) and trampoline fractures (66%), both relatively common paediatric fracture types that occur within a specific age group and present with distinct clinical features. Both fracture types are often quite subtle and difficult to detect [10, 11].

In the elbow joint, the PPV for fracture detection was low (69%), when all cases with a confidence score between 50% and 90% (doubt) were considered positive fracture detection. Conversely, categorising doubt cases as negative fracture detection led to an increase in PPV (93%) but a notable drop in sensitivity, decreasing from 91.5% to 80.5%. As an occult fracture is defined to be invisible on conventional radiographs and may only be detectable through indirect fracture signs such as joint effusion or on follow-up images by signs of fracture healing [12]; the annotation “doubt” is particularly confusing in the elbow considering the relatively high frequency of occult fractures in that location. The only previous study which evaluated BoneView in the setting of an external assessment did not report the confidence scores [8]. In our study, the detection rate in the elbow was only high for complete supracondylar and complete radial neck fractures, with a relatively higher proportion of doubt cases per fracture location (21.7%) when compared to the forearm (4.1%) and lower leg (2.6%) fracture sites.

The possibility of detecting elbow joint effusion as a posttraumatic change on lateral conventional radiographs was first described by Norell in 1954 [13]. The detection of joint effusions on conventional radiographs is crucial for clinicians as it directly influences treatment decisions as an indicator of a possible occult fracture [14]. In our study, the overall detection of joint effusion in the elbow joint by the AI software was moderate (PPV 89.5%) and even lower in cases without an obvious fracture (PPV 78.8%). A previous study has evaluated the detection of elbow effusions in children using a deep learning algorithm with promising results [4]. However, that study had several limitations such as a limited training dataset and comparison of the performance to paediatric emergency clinicians rather than paediatric radiologists. In our study, the detection of dislocations in the elbow joint was low.

Three previous studies performed an external assessment of an AI aid for fracture detection. Nguyen et al. and Hayashi et al. assessed a dataset of 300 radiographs of the appendicular skeleton retrospectively using the software BoneView, which resulted in relatively small numbers of radiographs per anatomic region [6, 7]. The performance of the software was evaluated both as a stand-alone [7] and in collaboration with junior and senior radiologists [8]. Gasmi et al. and Dupuis et al. assessed the diagnostic accuracy of the AI-based software Rayvolve (AZmed, Paris, France), once in a test environment [15] and once including a large number of cases but spread across all anatomical regions [16]. All these studies have focused only on fracture detection without consideration of indirect fracture signs and dislocations. Timely detection of dislocations is of utmost clinical importance in order to avoid poor outcomes with limited function and chronic pain which may necessitate open reduction and corrective surgery [17].

One of the strengths of our study is the extensive dataset, allowing for a thorough evaluation of different fracture types and locations. Moreover, the fact that paediatric radiologists, who established the reference standard, were not blinded to the clinical information enhances the quality of the reference diagnoses.

There are some limitations to our study. As our assessment of the AI software was limited to the three most common paediatric fracture locations, the results cannot be generalized to other sites of the paediatric skeleton. The retrospective study design and single institution setting are other limitations. Furthermore, only a single commercial AI software was evaluated and the results are not generalizable to other AI software. Further studies looking at the performance of AI-fracture detection in a paediatric emergency department or paediatric practice are necessary for gauging its clinical impact.

In conclusion, our study underlines the potential of AI-based software applications for automated fracture detection in paediatric patients with promising results for forearm and lower leg radiographs. However, for specific paediatric fracture types such as bowing fractures, toddler’s fractures, and trampoline fractures, as well as fractures, indirect fracture signs (joint effusion) and dislocations in the elbow, improvements are mandatory before paediatric practitioners or emergency physicians can confidently rely on AI-based fracture detection in their clinical practice.

## Data Availability

The images analyzed for the current study are not publicly available due to personal data protection. An anonymized summary of the results is available from the corresponding author on reasonable request.
